# A Cross-Study Analysis for Reproducible Sub-classification of Traumatic Brain Injury

**DOI:** 10.3389/fneur.2018.00606

**Published:** 2018-08-13

**Authors:** Bing Si, Gina Dumkrieger, Teresa Wu, Ross Zafonte, David W. Dodick, Todd J. Schwedt, Jing Li

**Affiliations:** ^1^Department of Industrial Engineering and Computer Engineering, School of Computing, Informatics, and Decision Systems Engineering, Arizona State University, Tempe, AZ, United States; ^2^Department of Neurology, Mayo Clinic, Phoenix, AZ, United States; ^3^Department of Physical Medicine and Rehabilitation, Harvard Medical School, Spaulding Rehabilitation Hospital, Massachusetts General Hospital, Brigham and Women's Hospital, Boston, MA, United States

**Keywords:** traumatic brain injury, concussion, diagnosis, classification, outcomes, head injury

## Abstract

**Objective:** To identify reproducible sub-classes of traumatic brain injury (TBI) that correlate with patient outcomes.

**Methods:** Two TBI datasets from the Federal Interagency Traumatic Brain Injury Research (FITBIR) Informatics System were utilized, Transforming Research and Clinical Knowledge in Traumatic Brain Injury (TRACK-TBI) Pilot and Citicoline Brain Injury Treatment Trial (COBRIT). Patients included in these analyses had closed head injuries with Glasgow Comas Scale (GCS) scores of 13–15 at arrival at the Emergency Department (ED). Sparse hiearchical clustering was applied to identify TBI sub-classes within each dataset. The reproducibility of the sub-classes was evaluated by investigating similarities in clinical variable profiles and patient outcomes in each sub-class between the two datasets, as well as by using a statistical metric called in-group proportion (IGP).

**Results:** Seven TBI sub-classes were identified in the first dataset. There were between-class differences in patient outcomes at 90 days (Glasgow Outcome Scale Extended (GOSE): *p* < 0.001) and 180 days (Trail Making Test (TMT): *p* = 0.03). Four of seven sub-classes were reproducible in the second dataset with very high IGPs (94, 100, 99, 97%). Seven TBI sub-classes were also identified in the second dataset. There were significant between-class differences in patient outcomes at 180 days (GOSE: *p* = 0.024; Brief Symptom Inventory (BSI) *p* = 0.007; TMT: *p* < 0.001). Three of seven sub-classes were reproducible in the second dataset with very high IGPs (100% for all).

**Conclusions:** Reproducible TBI sub-classes were identified across two independent datasets, suggesting that these sub-classes exist in a general population. Differences in patient outcomes according to sub-class assignment suggest that this sub-classification could be used to guide post-TBI prognosis.

## Introduction

Traumatic brain injury (TBI) severity is sub-classified into “mild,” “moderate,” and “severe” categories based upon Glasgow Coma Scale (GCS) scores (1). In addition to GCS, some classification systems also consider duration of loss of consciousness, amnesia, alteration of awareness, and imaging evidence of traumatic head injury (2). The sub-classification of TBI might be further refined if additional information that is available at the time of the initial post-TBI patient evaluation was utilized. Si et al. previously performed a study in 2018 that classified mild TBI patients into sub-classes using patients' gender, employment, marital status, use of alcohol, use of tobacco, history of neurologic disease, history of psychiatric disease, injury mechanism and a few additional characteristics collected during the Emergency Department (ED) visit including head CT results, diastolic and systolic blood pressure, and use of intravenous fluids (3). The sub-class assignment in this prior study predicted 90 and 180-day patient outcomes. However, the previous study focused on finding sub-classes from a single dataset, Transforming Research and Clinical Knowledge in Traumatic Brain Injury (TRACK-TBI) Pilot (4). Sub-classification is more clinically relevant if the sub-classes found in one patient population are reproducible in other patient populations.

The objectives of this study were to identify TBI sub-classes from a group of patients collected from one dataset and evaluate the reproducibility of the sub-classes in another dataset [TRACK-TBI Pilot and Citicoline Brain Injury Treatment Trial (COBRIT)] (5). If sub-classes exist in both datasets, it provides more evidence that such sub-classes exist in the general TBI population and thus might inform clinical practice.

## Methods

### Data

After obtaining appropriate approvals for accessing data, two TBI datasets were downloaded from the Federal Interagency Traumatic Brain Injury Research (FITBIR) Informatics System^1^, TRACK-TBI Pilot^2^ and COBRIT.

### Patient selection

We included patients with a GCS score of 13–15 at arrival at the ED and who had a closed head injury. Four hundred and seventy-eight out of 599 patients in TRACK-TBI Pilot, and 564 out of 1,213 patients in COBRIT met these inclusion criteria.

### Clinical variables

Based on clinical knowledge and literature review, 37 clinical variables potentially relevant for TBI sub-classification were included in our study (6–12) (Table 1). We only considered clinical variables shared by both datasets and for which less than 10% of patients had missing values, except for two variables that we hypothesized to be exceptionally important for sub-classifying patients: post-traumatic amnesia (PTA) duration and pupil reaction at ED arrival. To perform sub-classification on clinical variables, we imputed missing data for clinical variables in TRACK-TBI Pilot and COBRIT separately with a classic imputation method, Multivariate Imputation by Chained Equations implemented in the R package “mice” (13).

**Table 1:** Clinical variables included in analyses.

**Variable category**	**Variable name**	**Type**	**Values**
Demographics	Age	Ordinal	1–3: <30, 30–45, >45
	Gender	Binary	0–1: female, male
	Ethnicity	Binary	0–1: not Hispanic or Latino, Hispanic or Latino
	Education	Binary	0–1: college and grad, before high and high
	Employment	Binary	0–1: employed, unemployed/not working
	Marital status	Binary	0–1: married/living together/common law, not married/living together/ common law
Medical history	Alcohol use	Binary	0–1: no, yes
	Prior developmental disease	Binary	0–1: no, yes
	Prior psychiatric disease	Binary	0–1: no, yes
Current TBI mechanism	Injury mechanism–bike	Binary	0–1: no, yes
	Injury mechanism–pedestrian	Binary	0–1: no, yes
	Injury mechanism–motorcycle	Binary	0–1: no, yes
	Injury mechanism–motor	Binary	0–1: no, yes
	Injury mechanism–other person	Binary	0–1: no, yes
	Injury mechanism–fall	Binary	0–1: no, yes
	Injury mechanism–striking	Binary	0–1: no, yes
	Injury mechanism–other types	Binary	0–1: no, yes
ED examination	GCS total score at ED arrival	Numerical	13–15
	GCS assessment condition at ED arrival	Binary	0–1: no sedation or paralysis, sedation or paralysis
	GCS total score at ED discharge	Numerical	3–15
	Hospital type	Binary	0–1: primary, secondary
	Post-traumatic amnesia duration	Ordinal	1–3: none, < 1 min, ≥1 min
	CT result	Binary	0–1: without abnormality, abnormal
	Pupil reactivity at ED arrival	Ordinal	1–3: both, one, neither reactive
Blood work	Alcohol intoxication at ED	Binary	0–1: no, yes
	Any drug intoxication	Binary	0–1: no, yes
Vital signs	Diastolic blood pressure at ED	Ordinal	1–3: low (less than 60 mm Hg), normal (60–89 mm Hg), high (at least 90 mm Hg)
	Systolic blood pressure at ED	Ordinal	1–3: low (less than 90 mm Hg), normal (90–139 mm Hg), high (at least 140 mm Hg)
	Heart rate at ED	Ordinal	1–3: low (less than 60 bpm), normal (60–100 bpm), high (at least 101 bpm)
	Temperature at ED		1–3: low (less than 35 degrees Celsius), normal (35–37.7 degrees Celsius), high (greater than 37.7 degrees Celsius)
	O_2_ saturation at ED	Ordinal	1–2: low (less than 90%), normal (at least 90%)
	Respiratory rate at ED	Ordinal	1–3: low (less than 12/min), normal (12–20/min), high (at least 21/min)
Complications and treatment at ED	Blood transfusion	Binary	0–1: no, yes
	Hypotension	Binary	0–1: no, yes
	Hypoxia	Binary	0–1: no, yes
	Intubation	Binary	0–1: no, yes
	Seizure	Binary	0–1: no, yes

Of note, the definition of injury due to “other person” in TRACK-TBI Pilot was “injury purposefully inflicted by other persons.” “Primary” vs. “Secondary” hospital type is determined by “whether the participant/subject was taken directly from the scene of accident to the study hospital (primary) or was first taken to a non-study hospital, and then transferred to the study hospital (secondary).”

### Outcome variables

We included five patient outcome measures shared by TRACK-TBI Pilot and COBRIT to evaluate post-TBI global outcomes, psychological status, and cognitive activity limitations/neuropsychological impairment. Scores on each outcome measure were defined as consistent with “good” or “bad” outcomes. Specifically, a Glasgow Outcome Score Extended (GOSE) at 90 or 180 days was considered as a bad outcome if GOSE was from 1 to 7; a good outcome otherwise (14). Brief Symptom Inventory (BSI) score at 180 days was considered bad if its Global Severity Index T-score was >63 or two or more subscales >63; a good outcome, otherwise (15).

Wechsler Adult Intelligence Scale (WAIS) score at 180 days was considered as a bad outcome if the score was >=1 standard deviation below the mean; a good outcome, otherwise (16). Trail Making Test (TMT) score at 180 days was considered as a bad outcome if either of the age adjusted normalized times in A and B was >=1 standard deviation above mean; a good outcome, otherwise (17, 18).

### Sparse hierarchical clustering (SHC)

Hierarchical Clustering (HC) is a conventional clustering algorithm to build a binary tree that successively merges similar subgroups hierarchically. HC starts from the bottom of the tree where each subject is in its own subgroup and the pairwise distance between the subgroups is measured. In the next upper level of the tree, the pair of subgroups with the closest distance is merged into a bigger subgroup and the tree is iteratively built. However, since our study involved a large number of variables and not all of them contribute to sub-classifying patients, we used Sparse Hierarchical Clustering (SHC) (19), which automatically selects informative features to the clustering. SHC analysis was performed with an R package “sparcl.”

### Reproducibility of sub-classes in an independent dataset

Our objective was to evaluate the reproducibility of sub-classes identified within one dataset in another independent dataset, by classifying new patients into the sub-classes we found. First, we computed a centroid for each sub-class found in one dataset (dataset A), which averaged over the clinical variables of the patients within that sub-class. Then, we classified each patient in the second dataset (dataset B) to one sub-class in A by the shortest distance between the patient and the cluster-wise centroid. Note that it is possible that there exists a sub-class in A into which none of the patients in B is classified. Such a sub-class is definitely not reproducible. For the remaining sub-classes in A, i.e., sub-classes into which at least some patients in B were classified, we further assessed the reproducibility of these sub-classes using statistical criteria and medical criteria, both discussed below.

A cluster quality measure called “in group proportion (IGP)” was used to statistically assess the reproducibility of the sub-classes (20). IGP is between 0 and 1; the higher the IGP, the stronger evidence for the reproducibility of a sub-class. From the medical perspective, we assessed two aspects for reproducibility. First, if the average patient in a sub-class from the first dataset had similar measurements for his/her clinical variables to the average patient in a sub-class from the second dataset, there was evidence for the reproducibility of the sub-class. Similarities between the average patients was assessed by a Pearson correlation between their respective clinical variables. Second, we computed the proportions of bad outcomes among patients in sub-classes from each independent dataset, on each of the multi-dimensional outcome variables. A lack of difference in outcomes using a two-sample proportion test was further evidence for reproducibility.

## Results and discussion

Summary statistics for clinical variables in TRACK-TBI Pilot and COBRIT are shown in Table 2.

**Table 2:** Summary statistics of clinical variables included in the analysis.

**Variable category**	**Variable name**	**Summary statistics in TTP**	**Summary statistics in COBRIT**
Demographics	^*#^Age (< 30/30~45/>45)	155/107/216	164/115/285
	^*#^Gender (female/male)	138/340	162/402
	Ethnicity (not Hispanic or Latino/Hispanic or Latino)	403/75	535/29
	^*^Education (college and grad/before high and high)	148/330	113/451
	^*#^Employment (employed/unemployed)	275/203	386/178
	^*#^Marital status (married/not married)	151/327	232/332
Medical history	^*#^Alcohol use (no/yes)	238/240	219/345
	Prior developmental disease (no/yes)	424/54	503/61
	^*^Prior psychiatric disease (no/yes)	330/148	445/119
Current TBI mechanism	Injury mechanism—bike (no/yes)	398/80	505/59
	Injury mechanism—pedestrian (no/yes)	443/35	535/29
	Injury mechanism—motorcycle (no/yes)	465/13	477/87
	^#^Injury mechanism—motor (no/yes)	391/87	419/145
	Injury mechanism−other person (no/yes)	398/80	494/70
	^*#^Injury mechanism—fall (no/yes)	325/153	417/147
	Injury mechanism—striking (no/yes)	462/16	549/15
	Injury mechanism—other types (no/yes)	464/14	552/12
ED examination	GCS total score at ED arrival (13/14/15)	16/99/363	60/110/394
	GCS assessment condition at ED arrival (no sedation or paralysis/sedation or paralysis)	473/5	535/29
	GCS total score at ED discharge (3–12/13/14/15)	11/5/43/419	54/29/86/395
	^#^Hospital type (primary/secondary)	395/83	397/167
	^*#^Post-traumatic amnesia duration (none/ < 1 min/≥1 min)	197/32/249	161/0/403
	^*^CT result (without/with abnormality)	254/224	3/561
	Pupil reactivity at ED arrival (both/one/neither reactive)	470/6/2	561/2/1
Blood work	^#^Alcohol intoxication at ED (no/yes)	405/73	428/136
	^#^Any drug intoxication (no/yes)	451/27	419/145
Vital signs	Diastolic blood pressure at ED (low/normal/high)	0/389/89	75/440/49
	^*#^Systolic blood pressure at ED (low/normal/high)	0/318/160	0/387/177
	Heart rate at ED (low/normal/high)	11/395/72	10/466/88
	Temperature at ED (low/normal/high)	12/457/9	3/536/25
	O_2_ saturation at ED (low/normal/high)	1/477/0	12/552/0
	Respiratory rate at ED (low/normal/high)	1/438/39	9/453/102
Complications and treatment at ED	Blood transfusion (no/yes)	466/12	480/84
	^#^Hypotension (no/yes)	469/9	315/249
	Hypoxia (no/yes)	456/22	523/41
	Intubation (no/yes)	466/12	564/0
	Seizure (no/yes)	470/8	563/1

Clinical variables found by SHC that significantly contribute to sub-classifying Track-TBI Pilot patients or COBRIT patients are indicated by “*” and “#,” respectively.

### Reproducibility study

Initially we identified sub-classes using the TRACK-TBI Pilot data and validated the sub-classes using the COBRIT data. Then, we reversed the analysis and identified sub-classes from the COBRIT data and used TRACK-TBI Pilot data for validation. Variables that significantly contributed to patient sub-classification in TRACK-TBI Pilot and COBRIT were found by SHC and indicated with “^*^” and “^#^” in Table 2, respectively. Finally, we examined the similarity between the two sets of reproducible sub-classes found in the two datasets.

#### Reproducibility study of track-TBI pilot sub-classes using COBRIT

##### Sub-classification based on clinical variables in TRACK-TBI pilot

**Figure 1:**
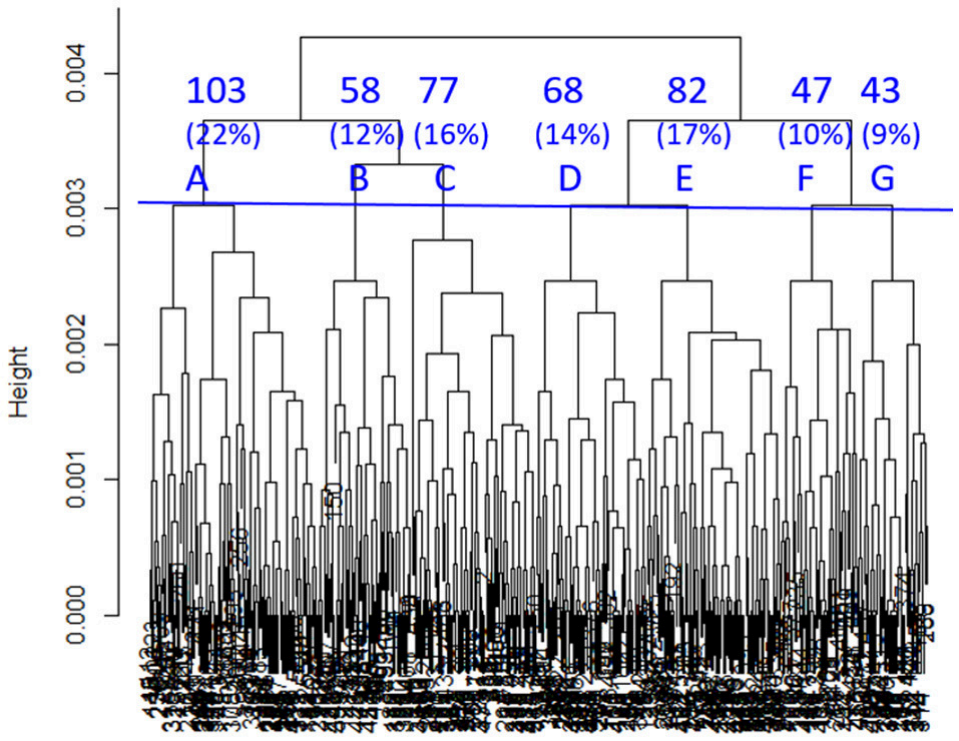
Dendrogram built by SHC. Seven clusters (A–G) were used in subsequent analyses. The percentages refer to the proportions of TRACK-TBI Pilot patients in each cluster.

SHC identified seven sub-classes with 11 clinical variables indicated by “^*^” in Table 2. As shown in Figure 1, sub-classes A-G included 22, 12, 16, 14, 17, 10, and 9% of all the TRACK-TBI Pilot subjects, respectively.

##### Subgroup characterization using clinical variables in TRACK-TBI pilot

**Figure 2:**
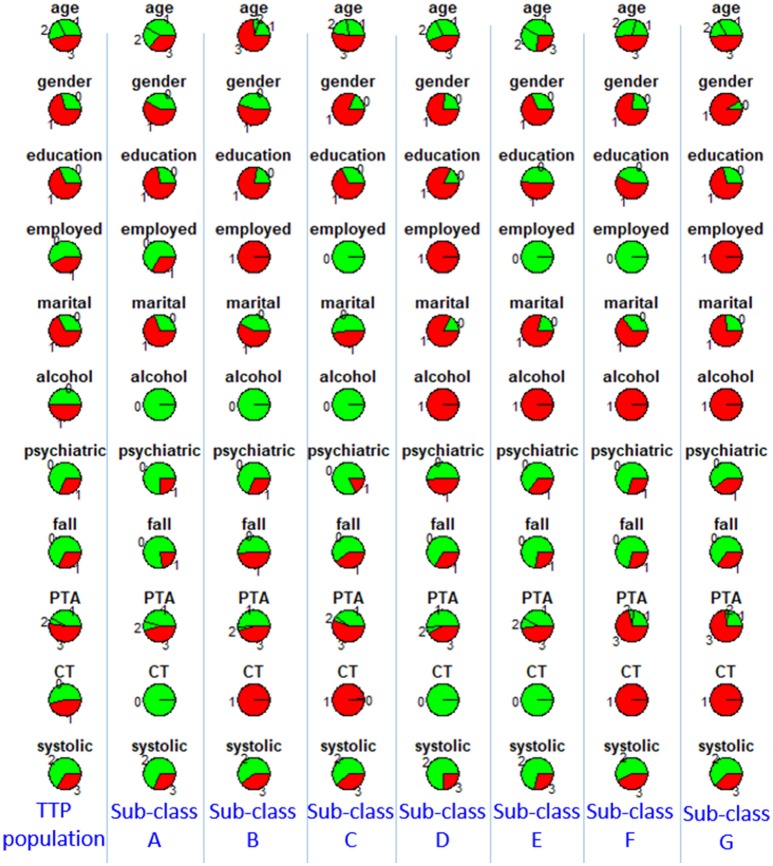
Distributions of clinical variables in clusters A–G and in the entire TRACK-TBI Pilot (TTP) population. Red represents proportions of age >45, male, education level being less than high school and high school, unemployment, unmarried, use of alcohol, having prior psychiatric disease, injury due to falls, post-traumatic amnesia >1 min, abnormal CT, abnormal systolic blood pressure at ED. Employed, employment; martial, marital status; alcohol, alcohol use; psychiatric, prior psychiatric disease; fall, injury mechanism being falls; PTA, post-traumatic amnesia; CT, CT result; systolic, systolic blood pressure at ED, TTP, Track-TBI Pilot.

To characterize the seven sub-classes using the 11 clinical variables, we used an 11 × 7 “pie chart array” in Figure 2 to show the distribution of each clinical variable within each sub-class. Colors of each pie represent the proportions of the different values for each variable. Please see Table 1 for the definition of “values” for each variable.

Furthermore, we explored the differences of multi-dimensional outcomes between sub-classes using an ANOVA test, which showed that there were significant between-class differences in GOSE at 90 days (*p* < 0.001) and TMT at 180 days (*p* = 0.03).

Then, to further investigate the between cluster differences in outcomes, we used a Chi-square test for proportions to compare GOSE at 90 days and TMT at 180 days between each pair of clusters. A few statistically significant ordinal relationships between a few pairs of sub-classes were identified (Table 3). For example, sub-class E had significantly better recovery in GOSE at 90 days, compared with sub-classes B, C, D, and G (*p* < 0.001, *p* < 0.001, *p* = 0.001, *p* < 0.001). Based on examination of the clinical profiles shown in Figure 2, we contend that this is because all patients in sub-class E had normal CT scans and were employed, and sub-class E contained more individuals with college or graduate degrees. We further compared outcomes between sub-classes B, C, D, and G, and found that sub-class C had better recovery in TMT at 180 days, compared with sub-classes D and G. This finding might be due to those in sub-class C not having a history of alcohol use.

**Table 3:** Pairwise comparison of outcomes between sub-classes.

	**E > B**	**E > C**	**E > D**	**E > G**	**C > D**	**C > G**
GOSE at 90 days	*p* < 0.001	*p* < 0.001	*p* = 0.001	*p* < 0.001		
GOSE at 180 days						
BSI at 180 days						
WAIS at 180 days						
TMT at 180 days					*p* = 0.005	*p* = 0.002

*P-values are shown only for those comparisons with significant differences*.

##### Validation of reproducibility using COBRIT

To validate if the sub-classes identified in TRACK-TBI Pilot were reproducible in COBRIT, we first classified each COBRIT patient into one of the seven sub-classes identified via analyses of the TRACK-TBI Pilot dataset. Sub-classes A-G included 2, 90, 142, 14, 6, 236, and 74 of the patients in COBRIT. Only 4% of COBRIT patients were classified into sub-classes A, D, and E. This is because A, D, and E in TRACK-TBI Pilot only included patients with normal CT while 99.5% of COBRIT patients had abnormal CT. Since 96% of COBRIT patients were classified into sub-classes B, C, F, and G, we focused on these four sub-classes in subsequent analyses. To measure the reproducibility of the four sub-classes in COBRIT, we computed the IGP for each sub-class. The IGPs of sub-classes B, C, F, and G were 94, 100, 99, and 97%, respectively. These IGPs are very high (close to the maximum possible value of 1), indicating very high reproducibility (20).

**Figure 3:**
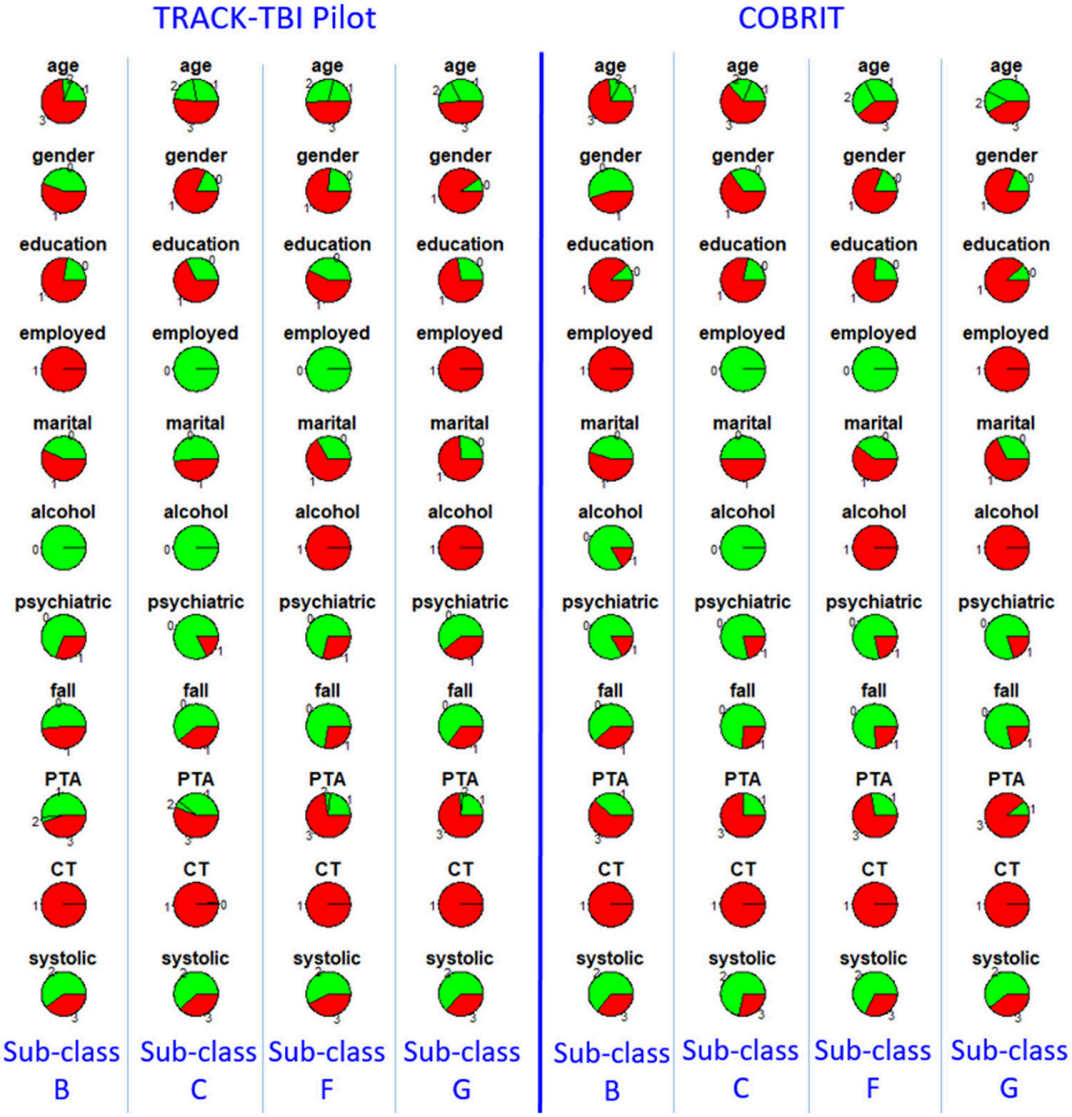
Distributions of clinical variables in four clusters B, C, F, and G in TRACK-TBI Pilot (left four columns) and in COBRIT patients who were classified into B, C, F, and G (right four columns). Colors and abbreviations are defined in the same way as Figure 2.

Furthermore, we checked reproducibility using clinical similarity criteria. In the left four columns of Figure 3, we show the pie chart array of sub-classes B, C, F, and G in TRACK-TBI Pilot (same as Figure 2). In the right four columns, we show the pie chart array of COBRIT patients who were classified into B, C, F, and G, respectively. Comparing the corresponding columns in the left and right panels, clear similarities can be seen. To quantify the similarity, we computed the Pearson correlation between the average patients of left-B and right-B sub-classes using their respective clinical variables. Likewise, we computed the Pearson correlation for other pairs of sub-classes. Correlations between sub-classes B, C, F, and G on the left pane and right pane are all larger than or equal to 0.99 (*p* < 0.001), providing evidence for the reproducibility of the sub-classes.

Another clinical criterion for reproducibility assessment is based on patient outcomes following TBI. Specifically, we tested the difference in each outcome between patients in the same sub-classes (B, C, F, or G) in TRACK-TBI Pilot and COBRIT. There were no significant differences in the proportion of patients experiencing bad outcomes between a TRACK-TBI Pilot sub-class and the COBRIT sub-class for each of the outcome variables. Since outcome variables were not used in SHC to produce the sub-classes, this result provides strong evidence that sub-classes B, C, F, and G found in TRACK-TBI Pilot were reproducible in COBRIT.

##### Prognosis of 180-day outcome using 90-day outcome

We pooled the patients from both datasets in each reproducible sub-class, and explored the differences of multi-dimensional outcomes between sub-classes using an ANOVA test, which showed that there was still significant between-class differences in GOSE (*p* = 0.03), BSI (*p* = 0.006), and TMT at 180 days (*p* = 0.009). Furthermore, we assessed the prognostic capability of 90-day GOSE for predicting 180-day GOSE in each sub-class, by computing the positive predictive value (PPV; proportion of patients with bad 90-day GOSE score who did not recover at 180 days) and negative predictive value (NPV; proportion of patients with good 90-day GOSE scores who did not deteriorate at 180 days) of each sub-class. For sub-classes B, C, F, and G, PPVs were 93.1, 77.5, 76.7, and 84.5%, respectively; NPVs were 71.9, 78.4, 81.7, and 87.5%, respectively, demonstrating clear differences between the sub-classes. Sub-class B had the highest PPV of 93.1%. The implication of this result is that if a patient is classified to sub-class B and has bad 90-day GOSE, he/she has 93.1% probability of having bad GOSE (i.e., non-recovering) at 180 days. Sub-class G had the highest NPV of 87.5%. This means that if a patient is classified to sub-class G and has good 90-day GOSE, he/she has 87.5% probability of having good GOSE (i.e., non-deteriorating) at 180 days. These probabilities would be different for the patient who is classified into any of the other sub-classes.

#### Reproducibility study of COBRIT sub-classes using TRACK-TBI pilot

##### Sub-classification based on clinical variables in COBRIT

**Figure 4:**
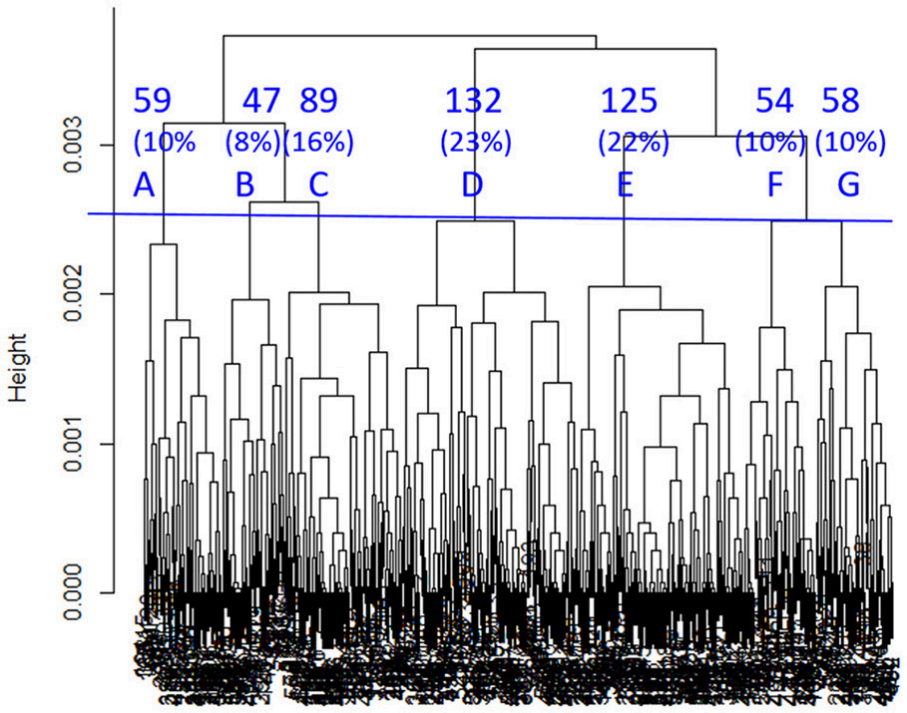
Dendrogram built by SHC. Seven clusters (A–G) were used in subsequent analyses. The percentages refer to the proportion of COBRIT patients in each cluster.

SHC identified seven sub-classes with 13 clinical variables indicated by “**#**” in Table 2. As shown in Figure 4, sub-classes A–G included 10, 8, 16, 23, 22, 10, and 10% of all the COBRIT subjects, respectively.

##### Subgroup characterization using clinical variables in COBRIT

**Figure 5:**
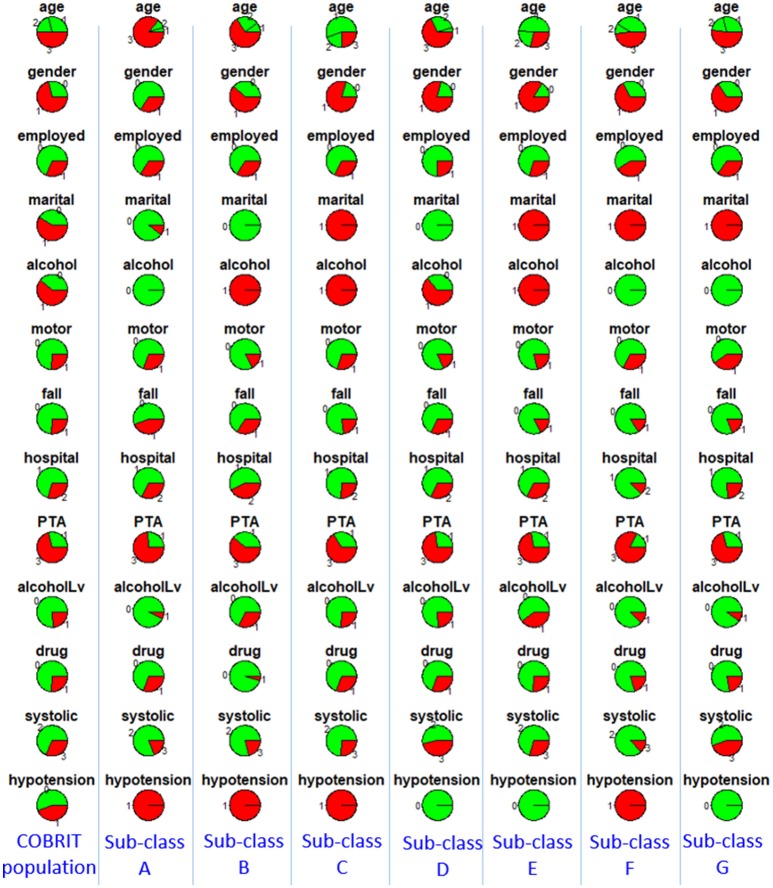
Distributions of clinical variables in clusters A–G and in the entire COBRIT population. Red represents proportions of age >45, male, unemployed, unmarried, use of alcohol, injury due to motor accidents, injury due to falls, primary hospital, post-traumatic amnesia >1 min, alcohol intoxication at ED, any drug intoxication at ED, abnormal systolic blood pressure at ED, hypotension presents at ED. Employed, employment; martial, marital status; alcohol, alcohol use; motor, injury mechanism being motor accident; fall, injury mechanism being falls; hospital, hospital type; PTA, post-traumatic amnesia; alcoholLV, alcohol intoxication; drug, any drug intoxication at ED; systolic, systolic blood pressure at ED; hypotension, hypotension present at ED.

To characterize the seven sub-classes using the 13 clinical variables, we used a 13 × 7 “pie chart array” in Figure 5 to show the distribution of each clinical variable within each sub-class.

Furthermore, we explored the differences of multi-dimensional patient outcomes between sub-classes using an ANOVA test, which showed that there were significant between-class differences in GOSE (*p* = 0.024), BSI (*p* = 0.007), and TMT (*p* < 0.001) at 180 days.

Then, to further investigate the between cluster differences in outcomes we used a Chi-square test for proportions to compare GOSE, BSI, and TMT at 180 days between each pair of clusters. A few statistically different ordinal relationships between a few pairs of sub-classes were identified (summarized in Table 4). For example, sub-class B had significantly better outcomes than sub-classes A, D, E, and G in terms of GOSE, BSI, and TMT at 180 days. Based on examination of the different clinical profiles between sub-classes (Figure 5), we hypothesize that superior outcomes amongst those in sub-class B might be due to a higher proportion of individuals being married, fewer patients having TBI due to motor vehicle accidents, and fewer patients with drug intoxication. Among other sub-classes, sub-class D had better recovery in TMT at 180 days compared to sub-classes E and G. This might be explained by differences in injury mechanisms; sub-class D includes fewer patients with injuries due to motor vehicle accidents and more patients with injuries due to falls.

**Table 4:** Pairwise comparison of outcomes between sub-classes.

	**B > A**	**B > D**	**B > E**	**B > G**	**C > G**	**D > E**	**D > G**
GOSE at 90 days							
GOSE at 180 days	*p* = 0.006	*p* = 0.006					
BSI at 180 days			*p* = 0.004	*p* < 0.001			
WAIS at 180 days							
TMT at 180 days				*p* = 0.002	*p* = 0.006	*p* = 0.001	*p* < 0.001

*P-values are shown only for those comparisons with significant differences*.

##### Validation of reproducibility using TRACK-TBI pilot

Since 99.5% of the patients in COBRIT had abnormal CT, we only included the TRACK-TBI Pilot patients with abnormal CT (224 patients) in the validation. We classified the 224 TRACK-TBI Pilot patients into one of the seven sub-classes found in COBRIT. Sub-classes A–G included 0, 1, 2, 90, 61, 2, and 68 patients in TRACK-TBI Pilot. Only 2% of TRACK-TBI Pilot patients were classified into sub-classes A, B, C, and F. One possible reason might be that A, B, C, and F in COBRIT contained patients with hypotension while 98.1% of TRACK-TBI Pilot patients had no hypotension. Since 97.8% of TRACK-TBI Pilot patients were classified into sub-classes D, E, and G, we focused on these three sub-classes in subsequent analyses. To measure the reproducibility of the three sub-classes in TRACK-TBI Pilot, we computed the IGP for each sub-class. The IGPs of sub-classes D, E, and G were each 100%, indicating high reproducibility.

**Figure 6:**
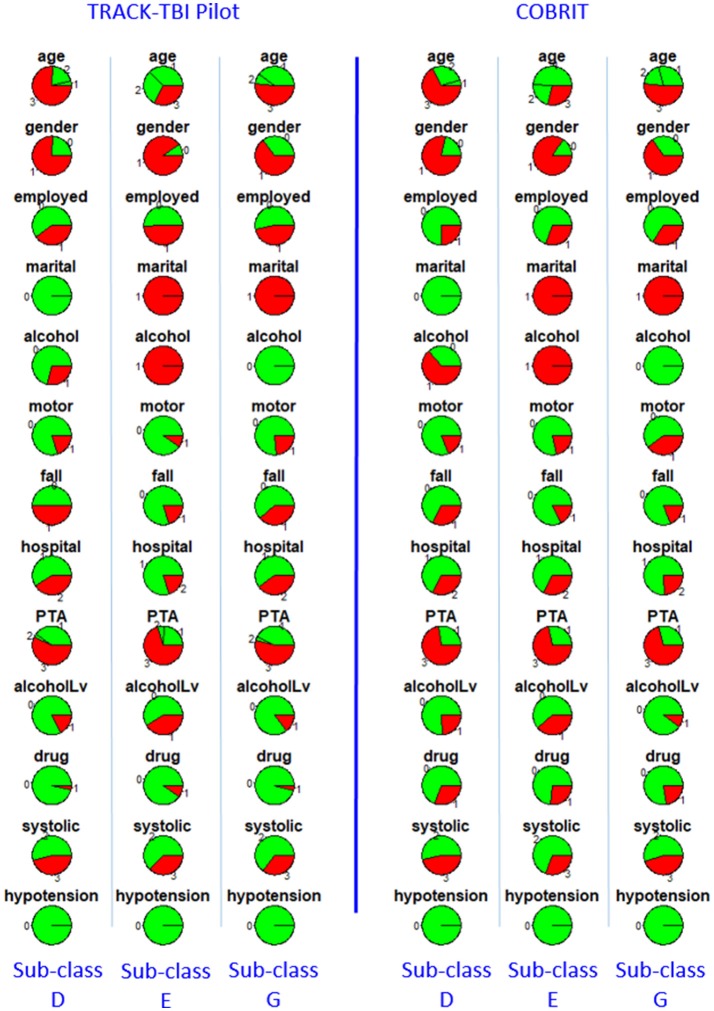
Distributions of clinical variables in three clusters D, E, and G for both TRACK-TBI Pilot patients who are classified into D, E, and G (left three columns) and COBRIT (right three columns). Colors and abbreviations are defined in the same way as Figure 5.

Furthermore, we checked reproducibility using clinical criteria by a pie chart array in Figure 6 (same as Figure 4). Comparing the corresponding columns in the left and right panels in Figure 6, we can see clear similarity. To quantify the similarity, we computed the Pearson correlation between the clinical variables of the average patients in sub-classes from the first dataset to the corresponding sub-class in the second dataset, and the correlations between sub-classes D, E, and G in two datasets are all larger than or equal to 0.99 (*p* < 0.001). All the correlations are near one, which is strong evidence for reproducibility.

We also tested the difference in each patient outcome between a sub-class (D, E, or G) in COBRIT and TRACK-TBI Pilot patients who were classified into that sub-class. There were no significant differences in the proportion of patients with bad outcomes within each sub-class between the two datasets, providing strong evidence for reproducibility.

To assess how the sub-classification results can help inform outcome prognosis we focused on sub-classes D, E, and G that were found in COBRIT and reproducible in TRACK-TBI Pilot. After pooling the patients from both datasets into each reproducible sub-class, ANOVA was used to investigate the differences in multi-dimensional patient outcomes between sub-classes. There were still significant between-class differences in TMT at 180 days (*p* < 0.001).

##### Prognosis of 180 days outcome using 90 days outcome

We assessed the prognostic capability of 90-day GOSE for predicting 180-day GOSE in each sub-class. For sub-classes D, E, and G, PPVs are 85.5, 76.3, and 80.6%, respectively; NPVs are 87.2, 82.9, and 71.4%, respectively, demonstrating clear differences between the sub-classes. Sub-class D has the highest PPV of 85.5% and highest NPV of 87.2%. The implication of this result is that if a patient is classified to sub-class D and has bad 90-day GOSE, he/she has 85.5% probability of having bad GOSE (i.e., not recovering) at 180 days. Also, if a patient is classified to sub-class D and has good 90-day GOSE, he/she has 87.2% probability of having good GOSE (i.e., not deteriorating) at 180 days. These probabilities would be different if the patient were classified into any other sub-class.

## Discussion and conclusions

The main findings of our study are that there are reproducible sub-classes amongst TBI patients with closed head injuries who present with a GCS score of 13–15 at arrival to the ED, and that the sub-classes are associated with 90 and 180-day patient outcomes. The reproducibility of the sub-classes across the two datasets, TRACK-TBI Pilot and COBRIT, suggests that these sub-classes likely exist in the general patient population.

### Literature review

To the best of our knowledge, there are not previously published reproducibility studies using TRACK-TBI Pilot and COBRIT datasets. However, Hart et al. (21) explored COBRIT data and identified race, age, PTA, employment status, and alcohol use history as significant predictors of 180-day BSI. In addition, there are studies using TRACK-TBI Pilot data to find multivariate predictive factors for post-TBI outcomes. Lingsma et al. (22) found that GOSE scores at 90 and 180 days were significantly associated with age, pre-existing psychiatric conditions, education years, injury caused by assault, and extracranial injury. Yuh et al. (23) discovered predictive factors for GOSE at 90 days including CT evidence of subarachnoid hemorrhage, unemployment, one or more brain contusions on MRI, and ≥4 foci of hemorrhagic axonal injury on MRI. Cnossen et al. (24) found that years of education, history of psychiatric disorders, and previous TBIs were the strongest predictors for RPQ at 180 days. Yue et al. (25) performed multivariate regression and found that alcohol intoxication at ED was associated with a higher odds-ratio of having GOSE ≤7 and a lower WAIS. Another study focusing on multivariate analysis of mild TBI patients showed that GOSE at 180 days was associated with clinical and demographics/socioeconomic variables, including education years, history of prior seizure, and GCS score at ED. Palacios et al. (26) compared 75 mild TBI patients from TRACK-TBI Pilot with 47 healthy subjects, and found that alterations in the spatial maps of brain resting-state functional connectivity networks between the two groups were predictive of RPQ, TMT, and CVLT at 180 days.

Nielson et al. (27) adopted a topological data analysis machine learning approach of TRACK-TBI Pilot data to discover patient subtypes according to clinical assessments, demographics, imaging and genetic findings, and clinical outcomes. This analytic approach identified a subgroup of TBI patients who had poorer outcomes, associated with higher rates of PTSD and single-nucleotide polymorphisms associated with DNA damage. Their study is different from ours in the following aspects: (1) their clustering structure was found from clinical variables and outcomes at 180 days, while our clustering structure was based on clinical variables available at the time of initial clinical evaluation; (2) subtypes found in their study were evaluated by correlating with genetic information, but the subtypes we discovered were evaluated by correlating with post-TBI outcomes at 90 and 180 days; (3) their study focused on a single dataset, while ours was a cross-study of two datasets to find reproducible sub-classes.

Consistent with the previous studies using TRACK-TBI Pilot or COBRIT data, our study also identified age, CT results, education, alcohol intoxication at ED, and psychiatric disease history as significant clinical variables contributing to TBI patient sub-classification. In addition, there are other contributing factors to sub-classifying TBI patient in our study, including gender, employment status, marital status, alcohol use history, TBI due to falling, TBI due to motor accidents, hospital type, PTA duration, drug use history, systolic blood pressure in the ED, and hypotension in the ED. Several of these factors have been investigated and some have been identified as predictors for patient outcomes in studies using datasets other than TRACK-TBI Pilot or COBRIT (28–30). Our study provides further evidence that a combination of multiple factors is associated with patient outcomes following TBI. These factors fit broadly into several categories, including sociodemographics, medical history, drug and alcohol use, mechanism of TBI, measures of TBI severity, and brain imaging findings. Mechanisms by which each of these variables associates with outcomes following TBI need to be further explored. Furthermore, the cross-validation of TBI sub-classes in our study, increases the likelihood of the sub-classes existing in TBI patient populations outside of TRACK-TBI Pilot and COBRIT and increases the likelihood of this sub-classification structure being useful for accurate prediction of patient outcomes, a goal that has historically been difficult to achieve (28).

### Limitations

There are several limitations of this study: (1) The study only included clinical variables shared by both datasets. Therefore, it is possible that additional clinical variables could contribute to TBI sub-classification, but were not considered in our study. For example, CT results were used as a binary variable (i.e., either “normal” CT or “abnormal” CT), while we were not able to include more detailed Rotterdam, Helsinki, or Stockholm CT scores since detailed CT readings were not available to us within the TRACK-TBI Pilot dataset. (2) The rules we used for binarizing outcomes into “good” vs. “bad” recovery were based on literature review and domain knowledge. Other approaches for binarization of the outcomes could be used. (3) The reproducible sub-classes were found and verified in two datasets only, i.e., TRACK-TBI Pilot and COBRIT. It is possible that there are other sub-classes yet to be discovered within a wider TBI population. (4) COBRIT was a TBI treatment trial with citicoline. Although the use of citicoline did not result in improvement in TBI outcomes compared with placebo (31), citicoline may have had some impact on patients in the active treatment group, potentially affecting the results of our analyses.

## Conclusions and future works

In conclusion, our study investigated the sub-classification of TBI amongst those with closed head injuries who had GCS scores of 13–15 at arrival to the ED. We studied two FITBIR datasets, TRACK-TBI Pilot and COBRIT, and independently found seven sub-classes from each dataset. Four of the seven sub-classes found in TRACK-TBI Pilot were reproducible in COBRIT and three of the seven sub-classes found in COBRIT were reproducible in TRACK-TBI Pilot. Furthermore, we used GOSE at 90 days to predict GOSE at 180 days, and showed the accuracy of the prognosis varied across different TBI sub-classes. Our study helps to identify the heterogeneity of TBI, even amongst patients all presenting with GCS scores between 13 and 15. This heterogeneity needs to be defined in order to accurately predict patient outcomes and to determine the most appropriate patient groups for TBI clinical trials.

If the sub-classes found in TRACK-TBI Pilot and COBRIT are proven to exist in the wider patient population, the sub-classes can help clinicians with their prognostication of each patient in the following way: A clinical variable profile for each sub-class (called sub-class-specific profile) can be computed by averaging over the clinical variables of the patients within that sub-class. Then, the clinician can compare a new patient's clinical variable profile with each of the sub-class-specific profiles and classify the new patient to the sub-class with the most similar profile. If further validated, this sub-classification can then be used to guide the prognosis of the patient with TBI.

Future TBI sub-classification work will include data collected over a broader time range, such as data collected at patient discharge in addition to data collected at the time of the initial patient evaluation. For example, including where the patient was discharged to (e.g., home, ward, or NCCU) would help take treatment intensity into account for the sub-classification. Other sub-classification studies will include additional datasets, helping to further define the reproducibility of the TBI sub-classes identified herein. Such studies are likely to provide additional insights that can be used for prognosticating patient outcomes following TBI.

## Author contributions

All authors contributed to the design of the work, data analyses, and/or interpretation of the data. All authors contributing to drafting the manuscript and/or revising it critically. All authors provided final approval of the submitted manuscript. All authors are accountable for all aspects of the work.

### Conflict of interest statement

The authors declare that the research was conducted in the absence of any commercial or financial relationships that could be construed as a potential conflict of interest.

## Footnotes

^1^“Federal Interagency Traumatic Brain Injury Research (FITBIR) Informatics System,” 2015, fitbir.nih.gov.

^2^“Transforming Research and Clinical Knowledge in Traumatic Brain Injury Pilot,” 2014, tracktbi.ucsf.edu.
